# Prediction of In-Hospital Cardiac Arrest in the Intensive Care Unit: Machine Learning–Based Multimodal Approach

**DOI:** 10.2196/49142

**Published:** 2024-07-23

**Authors:** Hsin-Ying Lee, Po-Chih Kuo, Frank Qian, Chien-Hung Li, Jiun-Ruey Hu, Wan-Ting Hsu, Hong-Jie Jhou, Po-Huang Chen, Cho-Hao Lee, Chin-Hua Su, Po-Chun Liao, I-Ju Wu, Chien-Chang Lee

**Affiliations:** 1 Department of Medicine, College of Medicine, National Taiwan University, Taipei, Taiwan; 2 Department of Computer Science, National Tsing Hua University, Hsinchu, Taiwan; 3 Section of Cardiovascular Medicine, Boston Medical Center, Boston, MA, United States; 4 Section of Cardiovascular Medicine, Boston University Chobanian & Avedisian School of Medicine, Boston, MA, United States; 5 Department of Internal Medicine, Yale School of Medicine, New Haven, CT, United States; 6 Department of Epidemiology, Harvard T.H. Chan School of Public Health, Boston, MA, United States; 7 Department of Neurology, Changhua Christian Hospital, Changhua, Taiwan; 8 Department of Internal Medicine, Tri-Service General Hospital, National Defense Medical Center, Taipei, Taiwan; 9 Division of Hematology and Oncology Medicine, Department of Internal Medicine, Tri-Service General Hospital, National Defense Medical Center, Taipei, Taiwan; 10 Department of Emergency Medicine, National Taiwan University Hospital, No. 7, Zhongshan S Rd, Zhongzheng District, Taipei, 100886 0223123456, Taiwan; 11 Department of Information Management, Ministry of Health and Welfare, Taipei, Taiwan

**Keywords:** cardiac arrest, machine learning, intensive care, mortality, medical emergency team, early warning scores

## Abstract

**Background:**

Early identification of impending in-hospital cardiac arrest (IHCA) improves clinical outcomes but remains elusive for practicing clinicians.

**Objective:**

We aimed to develop a multimodal machine learning algorithm based on ensemble techniques to predict the occurrence of IHCA.

**Methods:**

Our model was developed by the Multiparameter Intelligent Monitoring of Intensive Care (MIMIC)–IV database and validated in the Electronic Intensive Care Unit Collaborative Research Database (eICU-CRD). Baseline features consisting of patient demographics, presenting illness, and comorbidities were collected to train a random forest model. Next, vital signs were extracted to train a long short-term memory model. A support vector machine algorithm then stacked the results to form the final prediction model.

**Results:**

Of 23,909 patients in the MIMIC-IV database and 10,049 patients in the eICU-CRD database, 452 and 85 patients, respectively, had IHCA. At 13 hours in advance of an IHCA event, our algorithm had already demonstrated an area under the receiver operating characteristic curve of 0.85 (95% CI 0.815‐0.885) in the MIMIC-IV database. External validation with the eICU-CRD and National Taiwan University Hospital databases also presented satisfactory results, showing area under the receiver operating characteristic curve values of 0.81 (95% CI 0.763-0.851) and 0.945 (95% CI 0.934-0.956), respectively.

**Conclusions:**

Using only vital signs and information available in the electronic medical record, our model demonstrates it is possible to detect a trajectory of clinical deterioration up to 13 hours in advance. This predictive tool, which has undergone external validation, could forewarn and help clinicians identify patients in need of assessment to improve their overall prognosis.

## Introduction

The prognosis of in-hospital cardiac arrest (IHCA) is poor as it represents the culmination of heterogeneous multi-organ dysfunction, with few treatments [1]. IHCA has an incidence of 9 to 10 per 1000 admissions and a mortality rate of 80%‐100% [2]. Therefore, clinical guidelines emphasize the urgent need for early identification of patients at risk for IHCA [3]. Early warning scores were developed to facilitate early identification of impending clinical deterioration and trigger rapid interventions [4]. However, many traditional early warning scores are limited by considerable variation in discrimination in different populations and are often not sufficiently sensitive [5].

Recent research indicates that the implementation of the electronic Cardiac Arrest Risk Triage (eCART) score has significantly decreased the incidence of IHCA at UChicago Medicine [6]. However, the inclusion of laboratory data in eCART substantially diminishes the practicality and immediacy of this scoring system. Moreover, other studies have reported that calculating the Modified Early Warning Score (MEWS) 0.5 hours before a cardiac arrest can significantly increase the survival-to-discharge rate in patients experiencing IHCA [7]. Nonetheless, a 0.5-hour lead time is often insufficient for a prompt reaction during a patient’s rapid deterioration. Given the continuously generated real-time information, such as vital signs, a time-varying model could be constructed for more timely and early identification of IHCA.

The aim of our study was to develop a recurrent neural network–based model using the electronic health records (EHRs) of a single tertiary medical center to predict incident IHCA. We hypothesized that variations in physiological parameters, evaluated in the context of known comorbidities, could help to predict incident cardiac arrest. We also aimed to validate the model in an independent cohort and compare it to a previous scoring system.

## Methods

### Ethics Approval

Given the retrospective study design, the Research Ethics Committee of the National Taiwan University Hospital (NTUH) approved this study (project approval 202206108RINB) and waived the requirement for obtaining informed consent.

### Data Source

Predictive models were developed using the Multiparameter Intelligent Monitoring of Intensive Care (MIMIC)–IV v0.4 database and were externally validated using the Electronic Intensive Care Unit Collaborative Research Database (eICU-CRD) v2.0 [8,9]. Pre-existing institutional review board approval was waived given the deidentified nature of this public data set (Massachusetts Institute of Technology: 0403000206; Beth Israel Deaconess Medical Center: 2001-P-001699/14) [8]. One author who completed the Collaborative Institutional Training Initiative examination (certificate 57186438 for author HJJ) obtained access to the database and performed the data extraction. To assess the performance of our model in practical applications, we collected clinical data from the electronic medical records of the NTUH, spanning from 2008 to 2018. To decrease patient heterogeneity and feature variability, we applied the same inclusion criteria and data processing workflow to the 3 databases. We extracted data on patients older than 20 years who were hospitalized in intensive care units (ICUs) for at least 24 hours. Patients were excluded if they were encoded with a deceased status but without an IHCA labeling defined as below. We employed 5-fold cross-validation in our training cohort, randomly dividing the data set into 5 equally sized subsets. Four of these folds (80% of the MIMIC-IV cohort) were used for training, while the remaining fold (20% of the MIMIC-IV cohort) was reserved for internal validation. Performance metrics were recorded for each iteration, resulting in five distinct performance scores. These scores were then averaged to derive a singular more robust performance estimate for the model. Finally, external validation was performed on the entire eICU-CRD cohort.

### Disease Outcome Ascertainment

In the MIMIC-IV cohort, patients were marked with IHCA if they were either labeled with a time-stamped database-specific procedure code (22,5466 cardiac arrest) or diagnosed with the *International Classiﬁcation of Diseases, Ninth Revision* (*ICD-9*), Procedure Coding System (PCS) code 9960 (cardiopulmonary resuscitation, not otherwise specified). Although the MIMIC-IV database contained both *ICD-9* and *International Statistical Classification of Diseases, Tenth Revision* (*ICD-10*) codes, we did not convert *ICD-9*-PCS code 9960 to the *ICD-10*-PCS code, as the most approximately equivalent indicated code 5A1.2012 (performance of cardiac output, single, manual) represented variable definitions. For the eICU-CRD cohort, patients were classified with IHCA if they either presented with a time-stamped database-specific procedure note indicating cardiopulmonary resuscitation or were administered epinephrine, either as a bolus of 1 mg/10 ml or an infusion rate of 30 mg/250 ml at 100 ml/hr, with an associated administration time. In both the MIMIC-IV and eICU-CRD cohorts, the control group was defined as patients who were not labeled as having experienced an IHCA or being deceased, and the reference time was set as the ICU discharge time. For IHCA patients with multiple labelings, we only selected the time of the first label as the reference time. The data collection method in the NTUH database involves identifying patients with specific *ICD* codes (*ICD-9* 427.5; *ICD-10* T46.2, 145.8, 146.9). Patients who have been diagnosed with the aforementioned codes followed by the initiation of cardiopulmonary resuscitation or bolus epinephrine injection will be classified as patients who experienced IHCA.

### Data Curation and Features Extraction

Two types of features were extracted: time-independent baseline features and time-varying physiologic readings from bedside monitors. Baseline features, which are variables registered at the time of admission, consisted of three types: (1) demographic information such as gender, age, ethnicity, type of ICU admission, and BMI; (2) chronic comorbidities, as identified by combined comorbidity score and Elixhauser Comorbidity Index [10,11]; (3) presenting illness, as identified by *ICD* codes for acute cardiac disease, respiratory insufficiency, sepsis, and potential reversible causes of cardiac arrest, popularly known as the *H*’s (hyperkalemia, hypokalemia, hypothermia, hypoxemia, hypovolemia, hydrogen ion, eg, acidosis) and *T*’s (spontaneous tension pneumothorax, thrombosis, cardiac tamponade) by resuscitation guidelines [12]. Physiologic readings, which consisted of 6 vital signs: heart rate (HR), respiratory rate, O_2_ saturation (SpO_2_), systolic blood pressure (sBP), diastolic blood pressure, and mean arterial pressure, were extracted on an hourly basis. For all patients, vital signs in the 24 hours prior to the reference time were recorded. To balance model utility with adequate accuracy, we only investigated the risk of cardiac arrest starting from 13 hours prior to the event. To overcome the time series’ irregularity, specific rules were applied to combine multiple vital signs in the same hour (Multimedia Appendix 1). The remaining missing values in vital signs were filled with the last observation carried forward method. To eliminate the misguidance of our imbalanced data set, we tested the two following remedies: synthetic minority oversampling technique (SMOTE) and near miss algorithm [13,14]. We employed SMOTE in the following training with a nearest neighbor interpolation of 1 as it yielded a better performance compared to the near miss algorithm (Figure S1 in Multimedia Appendix 1). After applying SMOTE, the numbers of IHCA patients and control patients were equal, signifying data balance.

### Model Development

**Figure 1.**
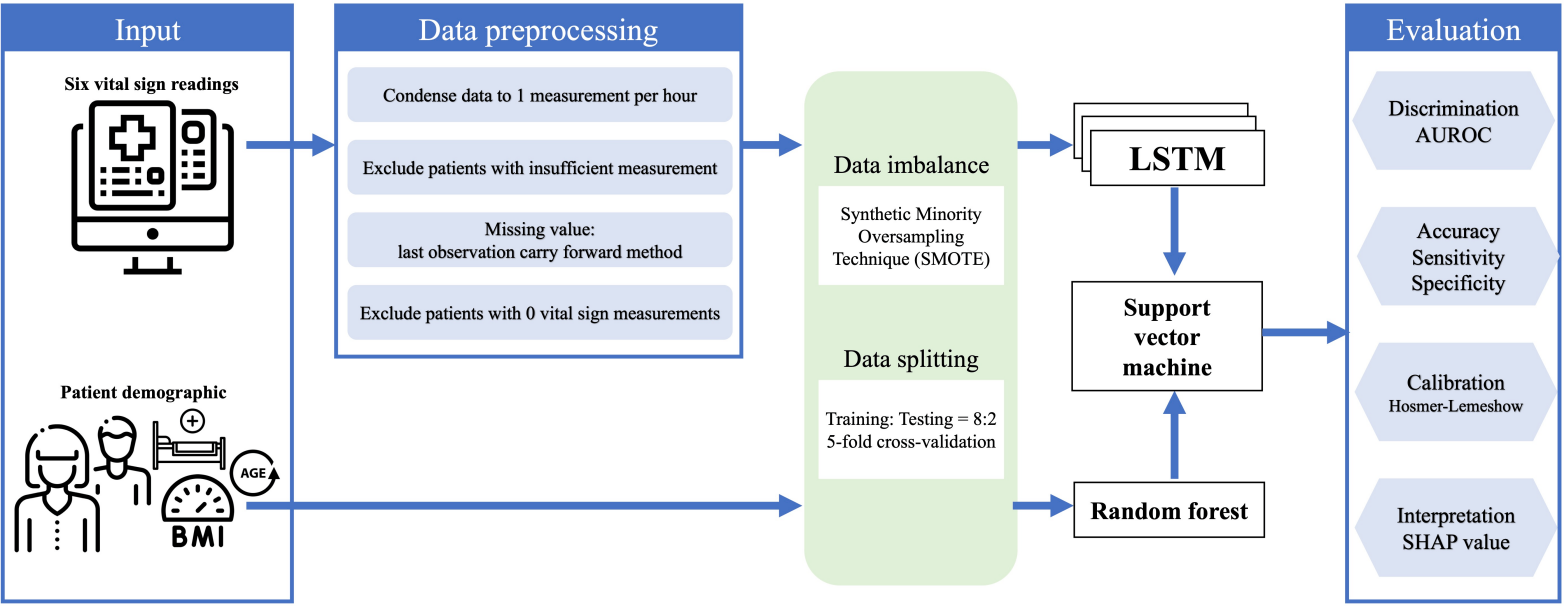
Illustration of the modeling framework. Each patient’s data from the electronic health record were used as input for our model. Four preprocessing steps are carried out on the vital signs to obtain fixed-interval data. All features go through SMOTE to overcome data imbalance and are split into training and testing groups. Baseline features are inputted to random forest, and vital signs are inputted into LSTM for prediction. Support vector machine then integrates both models. AUROC: area under the receiver operating characteristic curve; LSTM: long short-term memory; SHAP: Shapley Additive Explanations.

Our predictive model was encoded in three layers (Figure 1). First, random forest (RF) was responsible for classifying the baseline features [15]. For hyperparameter optimization, the number of estimators was set to 5, the maximum depth was set to 20, and Gini impurity was used to determine the split. Nodes are expanded until all leaves contain fewer than 2 samples [16]. Second, recurrent neural network with the long short-term memory (LSTM) architecture stored the vital signs trajectories in an hourly pattern [17]. There were 3 hidden layers and 8 cells each, with a tangent and a sigmoid activation function. The learning rate was set to 0.001, and a dropout rate of 0.4 was applied for regularization [18]. The Adam algorithm was adapted for optimizing network weights [19]. Last, the support vector machine (SVM) with a radial basis function kernel integrates the RF and LSTM models to generate the final prediction. The SVM predicts the identical target outcome by learning the relationship between the predictions from two base models (RF and LSTM) and the target outcomes in the training set [20]. All the models were implemented in Python 3.8.3 (Python Software Foundation) with TensorFlow 2.1.0, pandas 1.1.2, scikit-learn 0.24.2, and NumPy 1.19.1 libraries.

### Evaluation Strategy

To identify the perfect algorithm, the following machine learning (ML) techniques were evaluated in terms of prediction performance. First, based on the baseline data’s time independency and binary structure, logistic regression (LR), *k*-nearest neighbor (KNN), extreme gradient boosting (XGBoost) tree, and SVM were compared with RF for model fitness. In the LR model, we applied an L2 penalty with a stopping tolerance set at 1e-4, and the model underwent a maximum of 100 iterations. For the KNN algorithm, we set the parameter K to 2, utilizing Euclidean distance as the chosen metric. In the XGBoost model, the number of estimators was configured to 5 with a maximum depth of 5 and a learning rate of 0.1. Hyperparameter optimization was carried out through a grid search. In the SVM, we used a radial basis function with an L2 penalty, setting the regularization parameter to 1. The SVM model was executed with a stopping tolerance of 1e-3, and no limit was imposed on the maximum number of iterations. For the time-dependent vital signs trajectories, the incorporation of memory gates in LSTM indicates its superiority in handling long sequence data. Thus, no other model comparison was made. To compare different stacking techniques, LR was also implemented for comparison with SVM. Last, as we aim to use neural networks to accommodate our feature’s complexity, we connected this 3-layer model by engaging a deep neural network in baseline data prediction and final stacking. The hyperparameters of the deep neural network were set at an epoch of 30, batch size of 24, and the Adam algorithm as optimizer. Model performance was assessed based on discrimination and calibration using the internal validation cohort, as quantified by the area under the receiver operating characteristic curve (AUROC) with mean values and 95% CIs [21]. Sensitivity and specificity metrics are presented by two binary classifications, including a predefined threshold of 0.5 and an optimal cutoff determined by the Youden index [22]. We used the Brier score to assess accuracy and visualized calibration curves across deciles based on observed and expected cardiac arrest numbers [23].

### Model Interpretation

The importance of baseline features in the RF model was ranked based on “gain,” the cumulative improvement in accuracy of the nodes attributed to a specific feature. To focus more on the local impact of each vital sign at the patient level, we employed the Shapley Additive Explanations (SHAP) method to explain how our LSTM model makes predictions during a specific timepoint [24].

### Comparison With Previous Prediction Score

The Cardiac Arrest Risk Triage (CART), a commonly used cardiac arrest prediction model, was calculated to put the prediction results in perspective with prior studies [25]. A previously described “early warning score efficiency curve” was created to compare CART and our prediction model [26]. By plotting the percentage of detected events within 13 hours followed by the observations above the predefined threshold, a 0.5 probability in our model, and a score of 20 in the CART model, we could demonstrate the changes of cumulative incidence as the event time approached. Due to the large number of missing data for temperature and neurological status in our development cohort, we were unable to compare our risk prediction tool against the MEWS or Acute Physiology and Chronic Health Evaluation.

## Results

### Patient Characteristics

A total of 34,633 patients in the MIMIC-IV database and 79,643 patients in the eICU-CRD database were included in our analysis. After processing the vital signs data, a total of 452 IHCA patients and 23,457 control patients from MIMIC-IV were used for model development, whereas 85 IHCA patients and 9964 control patients from eICU-CRD were used for external validation. Table S1 in Multimedia Appendix 1 shows the baseline characteristics of the IHCA group and the control group for the two cohorts. IHCA patients were significantly older (*P*<.001) and scored higher on combined comorbidity scores and the Elixhauser Comorbidity Index. In terms of presenting illness, myocardial infarction, pneumonia, respiratory failure, and the 5 *H*’s and 5 *T*’s were more prevalent in IHCA patients than among control patients.

### Prediction From Time-Independent Data

**Figure 2.**
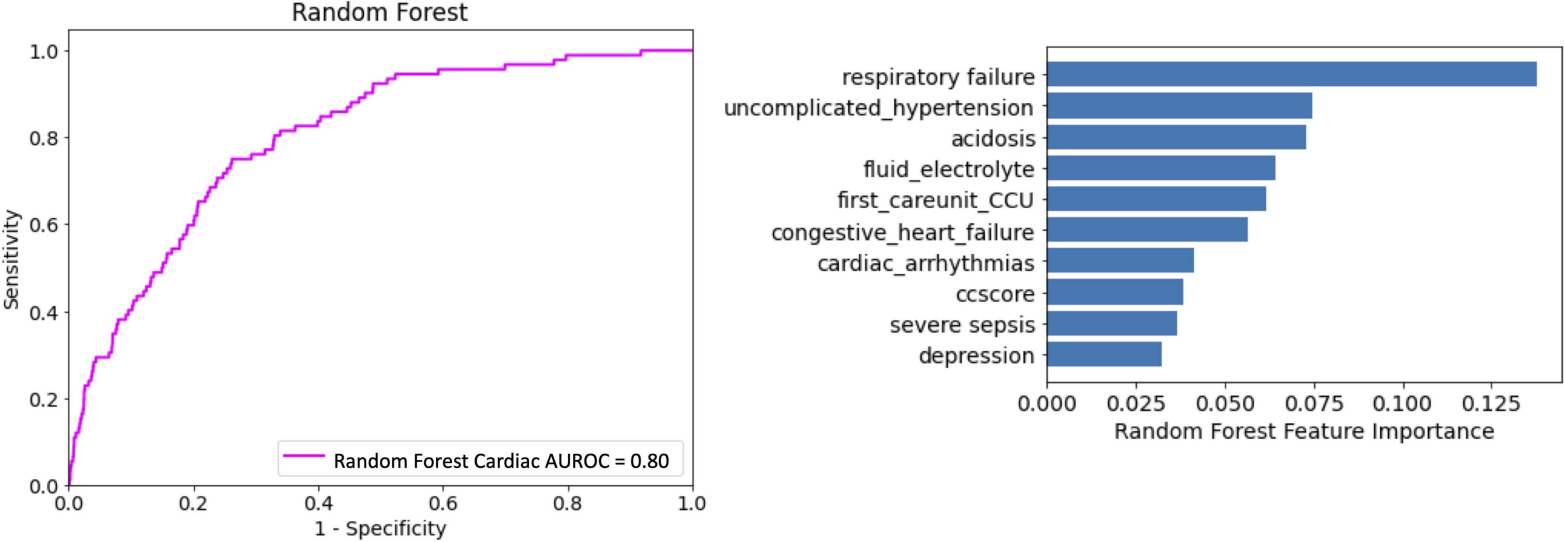
Prediction from baseline features. (A) AUROC for evaluating the discriminatory ability of random forest on baseline features. (B) Feature importance derived from the random forest model. AUROC: area under the receiver operating characteristic curve.

Patient demographics, comorbidities, and presenting illness were first classified by RF. Figure 2 demonstrates the discrimination of the RF model (AUROC 0.80, 95% CI 0.779‐0.844; sensitivity 0.71; specificity 0.78; *F*_1_-score 0.79. The top five important features listed by RF include the presence of respiratory failure or acidosis, comorbid uncomplicated hypertension, comorbid fluid and electrolyte disorder, and initial ICU being the cardiac ICU.

### Modeling of Time-Dependent Data

The trajectories of six vital signs were modeled with respect to time. Figure S2 A in Multimedia Appendix 1 illustrates that in the MIMIC-IV cohort, the control group exhibited a constant value of all six vital signs throughout the 24-hour collecting period. However, the vital signs of the IHCA patients were characterized by progressive deterioration in the last several hours. Of note, throughout the 24-hour monitoring period, patients who developed cardiac arrest exhibited, on average, a 12-mmHg lower sBP, 1.5% lower SpO_2_, and a 9-bpm higher resting HR compared to the control group. However, the exact timing of the start of deterioration could not be clearly marked on the plot. A similar vital signs trajectory was seen in the eICU-CRD cohort (Figure S2 B in Multimedia Appendix 1).

### Prediction From Time-Dependent Data

The hourly AUROC values for predicting cardiac arrest are presented in Figure S3 in Multimedia Appendix 1, which shows the results after SMOTE and cross-validation. A steady rise in AUROC was observed in the hours leading up to cardiac arrest with a sharp increase in the preceding 3 hours.

### Performance of the SVM-Based Stacking Model

**Figure 3.**
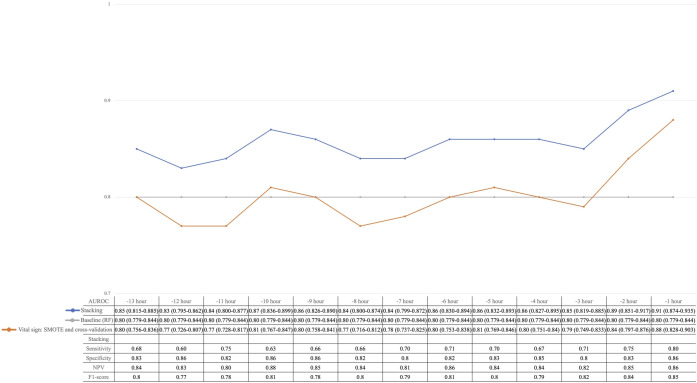
Performance of the stacked model in the Multiparameter Intelligent Monitoring of Intensive Care (MIMIC)–IV database. AUROCs (95% CIs) of the long short-term memory (LSTM) model with vital signs as input (orange plot), RF model with baseline features as input (gray plot), and stacked model after integration of RF and LSTM (blue plot) are shown. The three models’ exact AUROCs, sensitivity, specificity, NPV, and *F*_1_-score of the stacked model are listed in the table. AUROC: area under the receiver operating characteristic curve; NPV: negative predictive value; RF: random forest.

In the final step of model construction, we stacked the LSTM model with the RF model and combined both predictions from baseline features and vital signs. AUROCs of the stacked model exhibited consistently better predictions compared with the baseline and vital signs–only model, with the highest AUROC of 0.91 (95% CI 0.874‐0.935), sensitivity of 0.80, specificity of 0.86, and *F*_1_-score of 0.85 1 hour prior to the event. Further evaluation of the stacked model presented an increase in sensitivity, specificity, negative predictive value, and *F*_1_-score by the reduction of the time interval (Figure 3). However, the calibration plot showed a risk of overestimation and a steadily low Brier score throughout the 13 hours of prediction time (Figure S4 in Multimedia Appendix 1). Additionally, in Figure S5 in Multimedia Appendix 1, we compared the model performance using different cutoffs. We found that the optimal cutoff defined by the Youden index (at 13 hours: 0.29; at 12 hours: 0.25; at 11 hours: 0.38; at 10 hours: 0.25; at 9 hours: 0.28; at 8 hours: 0.26; at 7 hours: 0.28; at 6 hours: 0.30; at 5 hours: 0.26; at 4 hours: 0.38; at 3 hours: 0.30; at 2 hours: 0.34; at 1 hour: 0.35) presented with a better sensitivity compared with the predefined 0.5 cutoff; the largest difference was 14% at 12 hours prior to the event.

### External Validation

**Figure 4.**
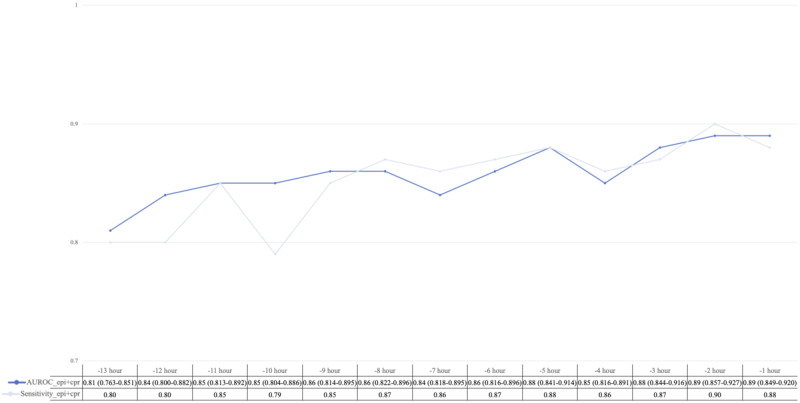
Performance of the stacked model in the Electronic Intensive Care Unit Collaborative Research Database (eICU-CRD). External validation of the stacked model is performed on the eICU-CRD. AUROC (95% CI) is plotted in a blue line; sensitivity is plotted in a gray line. AUROC: area under the receiver operating characteristic curve.

**Figure 5.**
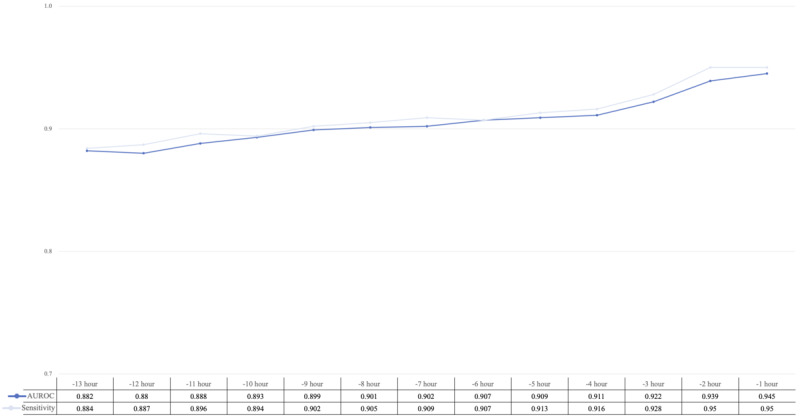
Performance of the stacked model in the clinical scenario. External validation of the stacked model is performed using data from 1935 in-hospital cardiac arrest patients and 3692 control patients collected from the National Taiwan University Hospital. AUROC is plotted in a blue line; sensitivity is plotted in a gray line. AUROC: area under the receiver operating characteristic curve.

We performed external validation of the stacked model in the eICU-CRD database. The results showed the best performance at 1 hour prior to IHCA with an AUROC of 0.89 (95% CI 0.849‐0.920), sensitivity of 0.79, specificity of 0.83, and an *F*_1_-score of 0.81. These findings align closely with the AUROC obtained from the MIMIC-IV data set (Figure 4). To further validate our model in an actual clinical scenario, we identified 1935 IHCA patients and 3692 control patients from the ICU of the NTUH. Additionally, our model demonstrated high prediction sensitivity and an AUROC of 0.945 when predicting IHCA 1 hour prior to its occurrence (Figure 5).

### Local Interpretation of the LSTM Model

We adopted the SHAP method to enable model explanation from an individual patient’s perspective. In each box, SHAP values for specific vital signs are assigned, with positive SHAP values in red indicating a risk factor and negative SHAP values in blue indicating a protective factor. Figure S6 A in Multimedia Appendix 1 represents a patient from the MIMIC-IV database experiencing IHCA at 0 hours. As IHCA approaches, an increase in sBP from its average contributes to an elevated risk, with the most significant effect occurring 6 hours prior to IHCA. However, at 1 hour before IHCA, the most significant risk becomes a decrease in sBP from its average. Figure S6 B in Multimedia Appendix 1 illustrates another IHCA patient from the eICU-CRD database. In contrast to Figure S6 A in Multimedia Appendix 1, the most prominent feature at 1 hour prior to IHCA is a decrease in HR and SpO_2_ from its baseline value. These figures showcase diverse presentations leading to IHCA in various patients, providing a valuable guideline for medical staff to identify the specific organ failure responsible for IHCA. The significance lies in enabling a swift response, incorporating timely interventions such as intubation for saturation drop and the administration of inotropic agents for decreased sBP. This approach ensures that medical staff will not delay necessary treatments while determining the cause of IHCA.

### Performance Compared With Different ML and Deep Learning Algorithms

Conventional statistics and supervised ML algorithms were compared to predict IHCA using only baseline features. RF demonstrated superior performance in terms of AUROC compared with XGBoost, LR, KNN, and SVM (Figure S7 in Multimedia Appendix 1). SVM also presented preferable results during the stacking operation compared with LR throughout the 13-hour prediction period. AUROCs at 1 hour prior to the incidence of IHCA were 0.91 versus 0.80 (Figure S8 in Multimedia Appendix 1). Finally, using a neural network to connect baseline, vital signs, and stacking predictions did not reveal an improving outcome (Figure S9 in Multimedia Appendix 1). After comparing several algorithms and combinations, RF, LSTM, and SVM predictions still yielded the most satisfactory results.

### Detection Efficacy Compared to Previous Prediction Score

We compared the performance of our proposed model to that of the CART score. Overall, our model demonstrated better AUROC throughout the prediction period (Figure S10 in Multimedia Appendix 1). As illustrated in Figure S11 in Multimedia Appendix 1, it is evident that at 12 hours prior to cardiac arrest, our model was able to detect over 70% of patients at risk for IHCA, compared to the CART score that did not surpass a 65% detection rate even 1 hour prior to IHCA.

## Discussion

### Principal Findings

In this retrospective study of 34,633 patients in the MIMIC-IV database, we constructed a high-performance multimodal model (AUROC 0.91, 95% CI 0.874‐0.935) that can predict IHCA up to 13 hours in advance using EHRs and high-resolution time series physiological readings. As the time of cardiac arrest approached, our model yielded a steady increase in the detection rate, finally reaching 89% 1 hour prior to the event. We also illustrated the impact of each vital sign on the prediction of cardiac arrest associated with individual patients through the use of SHAP values. Furthermore, we demonstrated the advantage of this ML algorithm over the CART score, which was derived using traditional regression models.

### Comparison to Prior Work

As a ubiquitous activity in the hospital, several studies have demonstrated the importance of vital signs measurement in determining a patient’s disease course [27]. Diastolic blood pressure, respiratory rate, and maximum HR have all been found to be significant and independent predictors of cardiac arrest [28]. However, maintaining a minimal model with only vital signs or adding lab data as predictors at the cost of decreasing model adaptability remains a dilemma [29,30]. The lactic acid level is the most representative laboratory biomarker in circulatory failure but had a high rate of missingness in the MIMIC-IV database (16,317/23,909, 68.2%). This motivated us to abandon utilizing lab results and assess if a nimbler model could be constructed with vital signs trends alone, overlaying the easily obtainable *ICD* codes and patient demographics as baseline features. Unsurprisingly, a significant increase in AUROC was discovered by adding demographics and comorbidities to the vital signs–only model. Furthermore, an SVM-based stacked model can address the predictive capabilities of underlying conditions and dynamic changes during disease deterioration. Stacking proves advantageous by compensating for the weaknesses of both models, with RF potentially struggling with highly correlated data and LSTM excelling in handling timely intricate information.

### Distinct Advantages of Our Approach

The reason for not establishing an end-to-end neural network throughout the prediction stood out, as supervised ML algorithms retained the ability to determine the importance of each predictor and have better model explainability. Moreover, in the ensemble technique, stacking excels over both boosting and bagging due to its versatility in integrating diverse data domains and combining various types of models. Late fusion at the model level is also preferred over other fusion methods for mitigating feature discrepancies and enabling independent model training between the time-independent baseline and time-dependent vital signs. Additionally, the outperformance of SVM over LR in the stacking operation could be attributed to better data handling using the nonlinear kernel function. To evaluate the external validity of our model, we tested it on two distinct data sets—the eICU-CRD and NTUH databases—both representing patient groups with diverse ethnicities and disease backgrounds. Over a 10-year duration, we identified 1935 (34.3%) IHCA cases in NTUH. In contrast to prior IHCA prediction studies, such as Kwon et al’s [31] 2.3% (n=1233) over 7 years, Chae et al’s [32] 1.3% (n=1154) over 4 years, and Ding et al’s [33] 23.09% over 5 years (n=1796), our clinical database demonstrated a higher IHCA incidence yet fewer cases [31-33]. This disparity is attributed to our ICU-focused validation database, in contrast to earlier studies that encompassed all patients who were hospitalized. Consequently, our approach ensures heightened data precision and a more nuanced understanding of patient dynamics through continuous monitoring within this critically ill cohort. Nevertheless, our high prediction quality in both independent databases ensures the credibility of our model across various demographic groups and subpopulations. The consistent performance across these data sets not only minimizes the possibility of overfitting but also validates the generalizability of our predictions.

### Limitations of Our Methodology

Our study had limitations because we used data collected from one medical center. First, due to the nature of EHRs, we were unable to determine the reason for the multi-scale gaps and different frequencies of each input. Second, we did not include clinical interventions, body temperature, and mental status in our model. Clinical interventions may change the disease course or even terminate the deterioration process. Nevertheless, the complexity of the treatment record and the high frequency of missing values in temperature and mental status compelled us to omit these valuable predictors. Third, our identification of IHCA relied on time-labeled database-specific procedure codes, *ICD* procedure codes, or administration of epinephrine in resuscitation dosages. In real-time clinical scenarios, delays in data entry may occur as documentation is considered secondary to patient care. Additionally, the accuracy of these codes is often operator dependent and may vary across different ICU policies. To minimize recording biases, we manually reviewed all IHCA vital signs data and only included reasonable measurements, ensuring that the identified IHCA timepoints correlated with the worst patient vital signs.

### Conclusion

We built a multimodal ML model based on time serial vital signs and three types of baseline features, which were all easily accessible in the ICU. Our model showed high accuracy in detecting clinical deterioration leading to the development of IHCA up to 13 hours in advance in both the internal and external validation cohorts. A model like this could be integrated into a hospital’s EHR system to identify high-risk patients and provide clinical decision support.

## Supplementary material

10.2196/49142 Multimedia Appendix 1 Supplementary tables, figures, and material.

## Abbreviations

AUROC: area under the receiver operating characteristic curve
CART: Cardiac Arrest Risk Triage
eCART: electronic Cardiac Arrest Risk Triage
EHR: electronic health record
eICU-CRD: Electronic Intensive Care Unit Collaborative Research Database
HR: heart rate
*ICD-9*: *International Classiﬁcation of Diseases, Ninth Revision*
*ICD-10*: *International Statistical Classification of Diseases, Tenth Revision*
ICU: intensive care unit
IHCA: in-hospital cardiac arrest
KNN: *k*-nearest neighbor
LR: logistic regression
LSTM: long short-term memory
MEWS: Modified Early Warning Score
MIMIC: Multiparameter Intelligent Monitoring of Intensive Care
ML: machine learning
NTUH: National Taiwan University Hospital
PCS: Procedure Coding System
RF: random forest
sBP: systolic blood pressure
SHAP: Shapley Additive Explanations
SMOTE: synthetic minority oversampling technique
SpO_2_: O_2_ saturation
SVM: support vector machine
XGBoost: extreme gradient boosting
