# Supplementary material for: Prediction of In-Hospital Cardiac Arrest in the Intensive Care Unit: Machine Learning–Based Multimodal Approach
Source: JMIR Med Inform. 2024 Jul 23;12:e49142. doi: 10.2196/49142 (PMC11287234; doi:10.2196/49142)
Supplement: Multimedia Appendix 1 [file medinform-v12-e49142-s001.docx]

**Machine Learning-Based Multimodal Prediction of In-Hospital Cardiac Arrest in the ICU**

^¶a^Hsin-Ying Lee MD, ^¶b^Po-Chih Kuo PhD, ^c,d^Frank Qian MD, MPH, ^b^Chien-Hung Li MS, ^e^Jiun-Ruey Hu MD, MPH, ^f^Wan-Ting Hsu MS, ^g^Hong-Jie Jhou MD, ^h^Po-Huang Chen MD, ^i^Cho-Hao Lee MD, ^j^Chin-Hua Su MSc, ^j^Po-Chun Liao MSc, ^j^I-Ju Wu MD, ^*j,k^Chien-Chang Lee MD, ScD

^a^Department of Medicine, College of Medicine, National Taiwan University, Taiwan

^b^Department of Computer Science, National Tsing Hua University, Hsinchu, Taiwan

^c^Department of Medicine, Beth Israel Deaconess Medical Center, Boston, MA, USA

^d^Department of Nutrition, Harvard T.H. Chan School of Public Health, Boston, MA, USA

^e^Department of Internal Medicine, Yale School of Medicine, USA

^f^Department of Epidemiology, Harvard T.H. Chan School of Public Health, Boston, MA, USA

^g^Department of Neurology, Changhua Christian Hospital, Changhua, Taiwan

^h^Department of Internal Medicine, Tri-Service General Hospital, National Defense Medical Center, Taipei, Taiwan

^i^Division of Hematology and Oncology Medicine, Department of Internal Medicine, Tri-Service General Hospital, National Defense Medical Center, Taipei, Taiwan

^j^Department of Emergency Medicine, National Taiwan University Hospital, Taipei, Taiwan

^k^Center of Intelligent Healthcare, National Taiwan University Hospital, Taipei, Taiwan

^¶^The two authors contribute equally to this work

***Correspondence to:**

Chien-Chang Lee, MD, ScD (Harvard)

Professor and Attending Physician, Department of Emergency Medicine, National Taiwan University Hospital

Principal Investigator, Health Data Science Research Group, National Taiwan University Hospital

Deputy Director, Center of Intelligent Healthcare, National Taiwan University Hospital

No.7, Chung Shan S. Rd., Zhongzheng Dist., Taipei City 100, Taiwan.

Email: hit3transparency@gmail.com/cclee100@gmail.com

TEL: +886-2-2312-3456 ext. 63485

**Supplementary Table:** 1

**Supplementary Figures:** 11

**Supplementary Material**

The multimedia appendix contains the following: 1. Supplementary Table: Descriptive statistics for the cohorts in MIMIC-IV and eICU-CRD databases. 2. Supplementary Figure: Performance of various machine learning models, including SMOTE, NearMiss, LSTM, stacked models, and Neural Networks, as well as vital sign trajectories and early warning score efficiency curves in the MIMIC-IV database. 3. Supplementary Material: Additional detailed descriptions of the methodology, data preprocessing steps, and analyses not included in the main manuscript.

**Supplementary Table**

**Supplementary Table 1: Descriptive statistics for the cohorts in MIMIC-IV and eICU-CRD database.**

|  | **MIMIC-IV** | | | **eICU-CRD** | | |
| --- | --- | --- | --- | --- | --- | --- |
|  | **Control** | **Cardiac arrest** | **p-value** | **Control** | **Cardiac arrest** | **p-value** |
| **Layer 1: Patient demographic** | | | | | | |
| Age | 63.02±15.61 | 65.48±15.01 | 0.001 | 64.16±13.99 | 59.49±15.38 | 0.002 |
| Gender |  |  | 0.973 |  |  | 0.630 |
| Male | 13786(58.8) | 266(58.8) |  | 3889(39.0) | 31(36.5) |  |
| Female | 9671(41.2) | 186(41.2) |  | 6075(61.0) | 54(63.5) |  |
| First admitted ICU |  |  | <0.001 |  |  | <0.001 |
| CCU | 2546(10.9) | 112(24.8) |  | 1563(15.7) | 4(4.7) |  |
| CVICU | 6594(28.1) | 75(16.6) |  | 323(3.2) | 6(7.1) |  |
| MICU | 3782(16.1) | 100(22.1) |  | 421(4.2) | 1(1.2) |  |
| MICU/SICU | 2920(12.4) | 44(9.7) |  | 3606(36.2) | 67(78.8) |  |
| Neuro ICU | 451(1.9) | 7(1.5) |  | 727(7.3) | 1(1.2) |  |
| CSICU/SICU/PACU | 3996(17.0) | 49(10.8) |  | 1795(18.0) | 3(3.5) |  |
| TSICU | 3168(13.5) | 65(14.4) |  | 1529(15.3) | 3(3.5) |  |
| Ethnicity |  |  | 0.007 |  |  | 0.022 |
| Native American | 41(0.2) | 1(0.2) |  | 73(0.7) | 0(0.0) |  |
| Asian | 653(2.8) | 9(2.0) |  | 151(1.5) | 2(2.4) |  |
| African American | 2008(8.6) | 59(13.1) |  | 994(10.0) | 12(14.1) |  |
| Hispanic/Latino | 870(3.7) | 15(3.3) |  | 314(3.2) | 9(10.6) |  |
| Unable to obtain | 361(1.5) | 9(2.0) |  | 86(0.9) | 0(0.0) |  |
| Unknown/other | 3505(14.9) | 81(17.9) |  | 453(4.5) | 3(3.5) |  |
| White | 16019(68.3) | 278(61.5) |  | 7893(79.2) | 59(69.4) |  |
| BMI |  |  | <0.001 |  |  | 0.466 |
| Missing | 9477(40.4) | 127(28.1) |  | 274(2.7) | 3(3.5) |  |
| Normal weight (18.5-24.9) | 3815(16.3) | 85(18.8) |  | 248(2.5) | 4(4.7) |  |
| Obese (>=30) | 5066(21.6) | 123(27.2) |  | 2373(23.8) | 20(23.5) |  |
| Overweight (25-29.9) | 4784(20.4) | 104(23.0) |  | 3142(31.5) | 22(25.9) |  |
| Underweight (<18.5) | 315(1.3) | 13(2.9) |  | 3927(39.4) | 36(42.4) |  |
| **Layer 2: Chronic comorbidity** | | | | | | |
| Combined comorbidity score | 2.19±2.50 | 3.92±2.89 | <0.001 | 0.29±0.95 | 1.35±1.93 | <0.001 |
| Congestive heart failure |  |  | <0.001 |  |  | 0.005 |
| No | 18539(79.0) | 256(56.6) |  | 9656(96.9) | 77(90.6) |  |
| Yes | 4918(21.0) | 196(43.4) |  | 308(3.1) | 8(9.4) |  |
| Arrhythmias |  |  | <0.001 |  |  | <0.001 |
| No | 15251(65.0) | 193(42.7) |  | 9508(95.4) | 67(78.8) |  |
| Yes | 8206(35.0) | 259(57.3) |  | 456(4.6) | 18(21.2) |  |
| Valvular heart disease |  |  | <0.001 |  |  | 0.267 |
| No | 20863(88.9) | 371(82.1) |  | 9746(97.8) | 85(100.0) |  |
| Yes | 2594(11.1) | 81(17.9) |  | 218(2.2) | 0(0.0) |  |
| Pulmonary circulation disease |  |  | <0.001 |  |  | 1.000 |
| No | 21747(92.7) | 368(81.4) |  | 9937(99.7) | 85(100.0) |  |
| Yes | 1710(7.3) | 84(18.6) |  | 27(0.3) | 0(0.0) |  |
| Peripheral vascular disease |  |  | <0.001 |  |  | 1.000 |
| No | 21479(91.6) | 387(85.6) |  | 9846(98.8) | 84(98.8) |  |
| Yes | 1978(8.4) | 65(14.4) |  | 118(1.2) | 1(1.2) |  |
| Uncomplicated hypertension |  |  | <0.001 |  |  | 0.508 |
| No | 13573(57.9) | 320(70.8) |  | 9054(90.9) | 79(92.9) |  |
| Yes | 9884(42.1) | 132(29.2) |  | 910(9.1) | 6(7.1) |  |
| Complicated hypertension |  |  | <0.001 |  |  | 1.000 |
| No | 19814(84.5) | 313(69.2) |  | 9958(99.9) | 85(100.0) |  |
| Yes | 3643(15.5) | 139(30.8) |  | 6(0.1) | 0(0.0) |  |
| Paralysis |  |  | 0.360 |  |  | 1.000 |
| No | 22563(96.2) | 431(95.4) |  | 9956(99.9) | 85(100.0) |  |
| Yes | 894(3.8) | 21(4.6) |  | 8(0.1) | 0(0.0) |  |
| Other neurological disorder |  |  | <0.001 |  |  | <0.001 |
| No | 20743(88.4) | 350(77.4) |  | 9809(98.4) | 75(88.2) |  |
| Yes | 2714(11.6) | 102(22.6) |  | 155(1.6) | 10(11.8) |  |
| Chronic pulmonary disease |  |  | <0.001 |  |  | 0.212 |
| No | 18567(79.2) | 312(69.0) |  | 9631(96.7) | 80(94.1) |  |
| Yes | 4890(20.8) | 140(31.0) |  | 333(3.3) | 5(5.9) |  |
| Diabetes without chronic complication |  |  | 0.227 |  |  | 0.002 |
| No | 19647(83.8) | 369(81.6) |  | 9907(99.4) | 81(95.3) |  |
| Yes | 3810(16.2) | 83(18.4) |  | 57(0.6) | 4(4.7) |  |
| Diabetes with chronic complication |  |  | 0.002 |  |  | 0.010 |
| No | 21206(90.4) | 389(86.1) |  | 9948(99.8) | 83(97.6) |  |
| Yes | 2251(9.6) | 63(13.9) |  | 16(0.2) | 2(2.4) |  |
| Hypothyroidism |  |  | 0.067 |  |  | 0.326 |
| No | 20786(88.6) | 388(85.8) |  | 9828(98.6) | 83(97.6) |  |
| Yes | 2671(11.4) | 64(14.2) |  | 136(1.4) | 2(2.4) |  |
| Renal failure |  |  | <0.001 |  |  | 0.007 |
| No | 19902(84.8) | 311(68.8) |  | 9494(95.3) | 75(88.2) |  |
| Yes | 3555(15.2) | 141(31.2) |  | 470(4.7) | 10(11.8) |  |
| Liver disease |  |  | <0.001 |  |  | <0.001 |
| No | 21303(90.8) | 358(79.2) |  | 9879(99.1) | 76(89.4) |  |
| Yes | 2154(9.2) | 94(20.8) |  | 85(0.9) | 9(10.6) |  |
| Peptic ulcer |  |  | 0.302 |  |  | 0.192 |
| No | 23251(99.1) | 446(98.7) |  | 9940(99.8) | 84(98.8) |  |
| Yes | 206(0.9) | 6(1.3) |  | 24(0.2) | 1(1.2) |  |
| AIDS |  |  | 0.649 |  |  | <0.001 |
| No | 23385(99.7) | 452(100.0) |  | 9964(100.0) | 85(100.0) |  |
| Yes | 72(0.3) | 0(0.0) |  |  |  |  |
| Lymphoma |  |  | 0.816 |  |  | 1.000 |
| No | 23209(98.9) | 447(98.9) |  | 9950(99.9) | 85(100.0) |  |
| Yes | 248(1.1) | 5(1.1) |  | 14(0.1) | 0(0.0) |  |
| Metastatic cancer |  |  | 0.634 |  |  | 1.000 |
| No | 22523(96.0) | 432(95.6) |  | 9930(99.7) | 85(100.0) |  |
| Yes | 934(4.0) | 20(4.4) |  | 34(0.3) | 0(0.0) |  |
| Solid tumor |  |  | 0.364 |  |  | 0.665 |
| No | 22827(97.3) | 443(98.0) |  | 9787(98.2) | 83(97.6) |  |
| Yes | 630(2.7) | 9(2.0) |  | 177(1.8) | 2(2.4) |  |
| Rheumatoid arthritis |  |  | 0.765 |  |  | 1.000 |
| No | 22636(96.5) | 435(96.2) |  | 9940(99.8) | 85(100.0) |  |
| Yes | 821(3.5) | 17(3.8) |  | 24(0.2) | 0(0.0) |  |
| Coagulopathy |  |  | <0.001 |  |  | <0.001 |
| No | 19848(84.6) | 311(68.8) |  | 9785(98.2) | 74(87.1) |  |
| Yes | 3609(15.4) | 141(31.2) |  | 179(1.8) | 11(12.9) |  |
| Obesity |  |  | 0.808 |  |  | 0.029 |
| No | 20690(88.2) | 397(87.8) |  | 9830(98.7) | 81(95.3) |  |
| Yes | 2767(11.8) | 55(12.2) |  | 134(1.3) | 4(4.7) |  |
| Weight loss |  |  | <0.001 |  |  | <0.001 |
| No | 22064(94.1) | 403(89.2) |  | 9864(99.0) | 75(88.2) |  |
| Yes | 1393(5.9) | 49(10.8) |  | 100(1.0) | 10(11.8) |  |
| Fluid electrolyte imbalance |  |  | <0.001 |  |  | <0.001 |
| No | 16109(68.7) | 177(39.2) |  | 9482(95.2) | 58(68.2) |  |
| Yes | 7348(31.3) | 275(60.8) |  | 482(4.8) | 27(31.8) |  |
| Blood loss anemia |  |  | 0.090 |  |  | 1.000 |
| No | 23135(98.6) | 450(99.6) |  | 9960(100.0) | 85(100.0) |  |
| Yes | 322(1.4) | 2(0.4) |  | 4(0.0) | 0(0.0) |  |
| Deficiency anemias |  |  | 0.455 |  |  | 1.000 |
| No | 22629(96.5) | 439(97.1) |  | 9962(100.0) | 85(100.0) |  |
| Yes | 828(3.5) | 13(2.9) |  | 2(0.0) | 0(0.0) |  |
| Alcohol abuse |  |  | 0.706 |  |  | 0.055 |
| No | 21312(90.9) | 413(91.4) |  | 9921(99.6) | 83(97.6) |  |
| Yes | 2145(9.1) | 39(8.6) |  | 43(0.4) | 2(2.4) |  |
| Drug abuse |  |  | 0.705 |  |  | 0.074 |
| No | 22329(95.2) | 432(95.6) |  | 9956(99.9) | 84(98.8) |  |
| Yes | 1128(4.8) | 20(4.4) |  | 8(0.1) | 1(1.2) |  |
| Psychoses |  |  | 0.873 |  |  | 1.000 |
| No | 23071(98.4) | 445(98.5) |  | 9956(99.9) | 85(100.0) |  |
| Yes | 386(1.6) | 7(1.5) |  | 8(0.1) | 0(0.0) |  |
| Depression |  |  | 0.028 |  |  | 0.094 |
| No | 19940(85.0) | 401(88.7) |  | 9905(99.4) | 83(97.6) |  |
| Yes | 3517(15.0) | 51(11.3) |  | 59(0.6) | 2(2.4) |  |
| **Layer 3: Presenting illness** | | | | | | |
| Coronary heart disease |  |  | 0.165 |  |  | 0.800 |
| No | 20687(88.2) | 389(86.1) |  | 9467(95.0) | 82(96.5) |  |
| Yes | 2770(11.8) | 63(13.9) |  | 497(5.0) | 3(3.5) |  |
| Myocardial infarction |  |  | <0.001 |  |  | 0.037 |
| No | 21199(90.4) | 354(78.3) |  | 9480(95.1) | 76(89.4) |  |
| Yes | 2258(9.6) | 98(21.7) |  | 484(4.9) | 9(10.6) |  |
| Cardiomegaly |  |  | 0.635 |  |  | <0.001 |
| No | 23394(99.7) | 452(100.0) |  | 9964(100.0) | 85(100.0) |  |
| Yes | 63(0.3) | 0(0.0) |  |  |  |  |
| Valvular heart disease |  |  | 0.257 |  |  | 0.442 |
| No | 20012(85.3) | 377(83.4) |  | 9481(95.2) | 83(97.6) |  |
| Yes | 3445(14.7) | 75(16.6) |  | 483(4.8) | 2(2.4) |  |
| Congenital heart disease |  |  | 0.152 |  |  | 1.000 |
| No | 22711(96.8) | 443(98.0) |  | 9950(99.9) | 85(100.0) |  |
| Yes | 746(3.2) | 9(2.0) |  | 14(0.1) | 0(0.0) |  |
| Ectopic rhythm disorder |  |  | 0.543 |  |  | 0.081 |
| No | 23417(99.8) | 451(99.8) |  | 9955(99.9) | 84(98.8) |  |
| Yes | 40(0.2) | 1(0.2) |  | 9(0.1) | 1(1.2) |  |
| Long QT syndrome |  |  | 0.303 |  |  | <0.001 |
| No | 23336(99.5) | 448(99.1) |  | 9964(100.0) | 85(100.0) |  |
| Yes | 121(0.5) | 4(0.9) |  |  |  |  |
| Pulmonary edema |  |  | 0.532 |  |  | <0.001 |
| No | 22794(97.2) | 437(96.7) |  | 9839(98.7) | 78(91.8) |  |
| Yes | 663(2.8) | 15(3.3) |  | 125(1.3) | 7(8.2) |  |
| Pneumonia |  |  | <0.001 |  |  | 0.001 |
| No | 20972(89.4) | 340(75.2) |  | 9571(96.1) | 74(87.1) |  |
| Yes | 2485(10.6) | 112(24.8) |  | 393(3.9) | 11(12.9) |  |
| Asthma AE |  |  | 0.970 |  |  | 0.630 |
| No | 22254(94.9) | 429(94.9) |  | 9835(98.7) | 85(100.0) |  |
| Yes | 1203(5.1) | 23(5.1) |  | 129(1.3) | 0(0.0) |  |
| COPD AE |  |  | 0.116 |  |  | 1.000 |
| No | 23061(98.3) | 440(97.3) |  | 9899(99.3) | 85(100.0) |  |
| Yes | 396(1.7) | 12(2.7) |  | 65(0.7) | 0(0.0) |  |
| Respiratory failure |  |  | <0.001 |  |  | <0.001 |
| No | 19612(83.6) | 198(43.8) |  | 8412(84.4) | 45(52.9) |  |
| Yes | 3845(16.4) | 254(56.2) |  | 1552(15.6) | 40(47.1) |  |
| Hyperkalemia |  |  | <0.001 |  |  | 0.001 |
| No | 22252(94.9) | 384(85.0) |  | 9848(98.8) | 79(92.9) |  |
| Yes | 1205(5.1) | 68(15.0) |  | 116(1.2) | 6(7.1) |  |
| Hypokalemia |  |  | 0.568 |  |  | 0.002 |
| No | 22501(95.9) | 436(96.5) |  | 9808(98.4) | 79(92.9) |  |
| Yes | 956(4.1) | 16(3.5) |  | 156(1.6) | 6(7.1) |  |
| Hypothermia |  |  | 1.000 |  |  | 0.058 |
| No | 23405(99.8) | 451(99.8) |  | 9958(99.9) | 84(98.8) |  |
| Yes | 52(0.2) | 1(0.2) |  | 6(0.1) | 1(1.2) |  |
| Hypoxemia |  |  | 0.538 |  |  | <0.001 |
| No | 22324(95.2) | 433(95.8) |  | 9734(97.7) | 72(84.7) |  |
| Yes | 1133(4.8) | 19(4.2) |  | 230(2.3) | 13(15.3) |  |
| Hypovolemia |  |  | 0.062 |  |  | <0.001 |
| No | 22602(96.4) | 428(94.7) |  | 9830(98.7) | 77(90.6) |  |
| Yes | 855(3.6) | 24(5.3) |  | 134(1.3) | 8(9.4) |  |
| Acidosis (Hydrogen ions) |  |  | <0.001 |  |  | <0.001 |
| No | 21088(89.9) | 301(66.6) |  | 9762(98.0) | 60(70.6) |  |
| Yes | 2369(10.1) | 151(33.4) |  | 202(2.0) | 25(29.4) |  |
| Spontaneous tension pneumothorax |  |  | 1.000 |  |  | 0.017 |
| No | 23450(100.0) | 452(100.0) |  | 9963(100.0) | 84(98.8) |  |
| Yes | 7(0.0) | 0(0.0) |  | 1(0.0) | 1(1.2) |  |
| Pulmonary embolism (Thrombosis) |  |  | <0.001 |  |  | 0.415 |
| No | 23134(98.6) | 435(96.2) |  | 9902(99.4) | 84(98.8) |  |
| Yes | 323(1.4) | 17(3.8) |  | 62(0.6) | 1(1.2) |  |
| Cardiac tamponade |  |  | 0.009 |  |  | 1.000 |
| No | 23249(99.1) | 442(97.8) |  | 9962(100.0) | 85(100.0) |  |
| Yes | 208(0.9) | 10(2.2) |  | 2(0.0) | 0(0.0) |  |
| Severe sepsis |  |  | <0.001 |  |  | 0.001 |
| No | 22415(95.6) | 369(81.6) |  | 9628(96.6) | 75(88.2) |  |
| Yes | 1042(4.4) | 83(18.4) |  | 336(3.4) | 10(11.8) |  |
| Septic shock |  |  | <0.001 |  |  | <0.001 |
| No | 22169(94.5) | 353(78.1) |  | 9618(96.5) | 69(81.2) |  |
| Yes | 1288(5.5) | 99(21.9) |  | 346(3.5) | 16(18.8) |  |

Data are presented as mean ± standard deviation or number (%).

**Supplementary Figure**

**Figure S1: Performance of the SMOTE and NearMiss method.**

AUROCs of LSTM model using vital signs that underwent SMOTE (orange plot) or NearMiss (blue plot) as the solution for data imbalance.

**
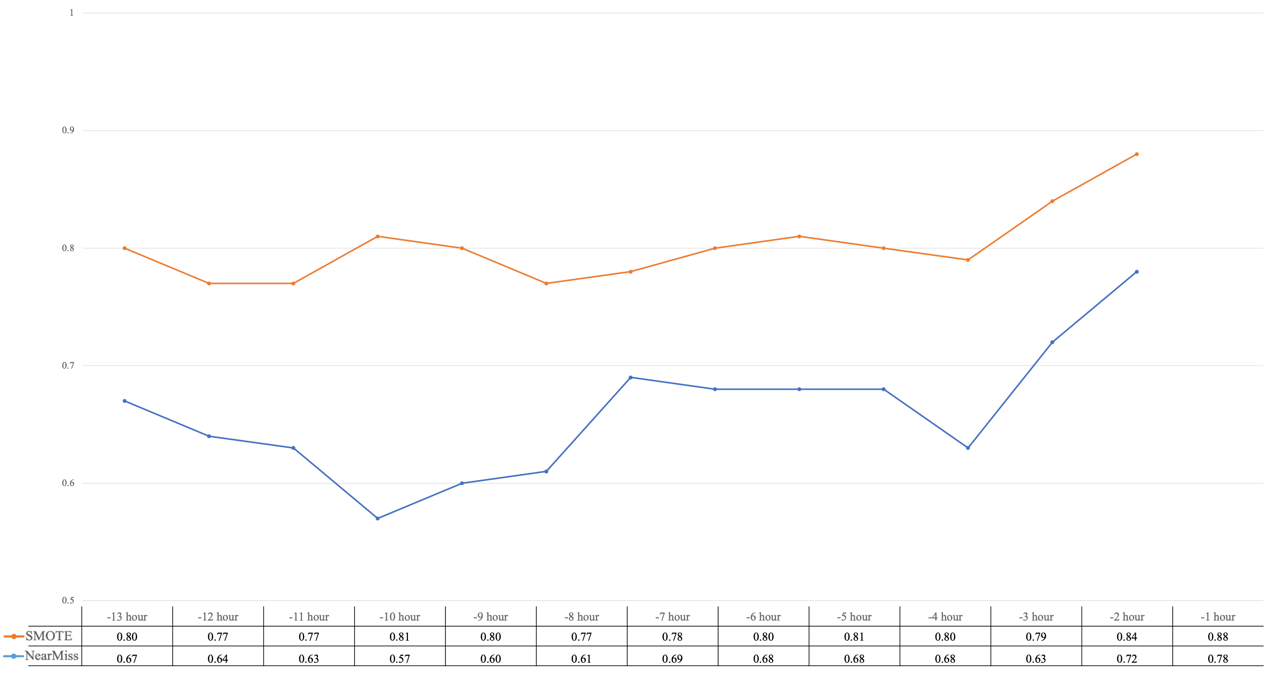
**

**Figure S2: Six vital sign trajectories in a prediction interval of 24 hours.**

(A) Mean of six vital signs of all patients in the MIMIC-IV database. The cardiac arrest group is presented in a yellow plot; the control group is presented in a gray plot. (B) Mean of six vital signs of all patients in the eICU-CRD database. The cardiac arrest group is presented as a green plot; the control group is presented as a gray plot. eICU-CRD, Electronic ICU Collaborative Research Database; MIMIC, Multiparameter Intelligent Monitoring of Intensive Care.

**Supplementary Figure 2A**
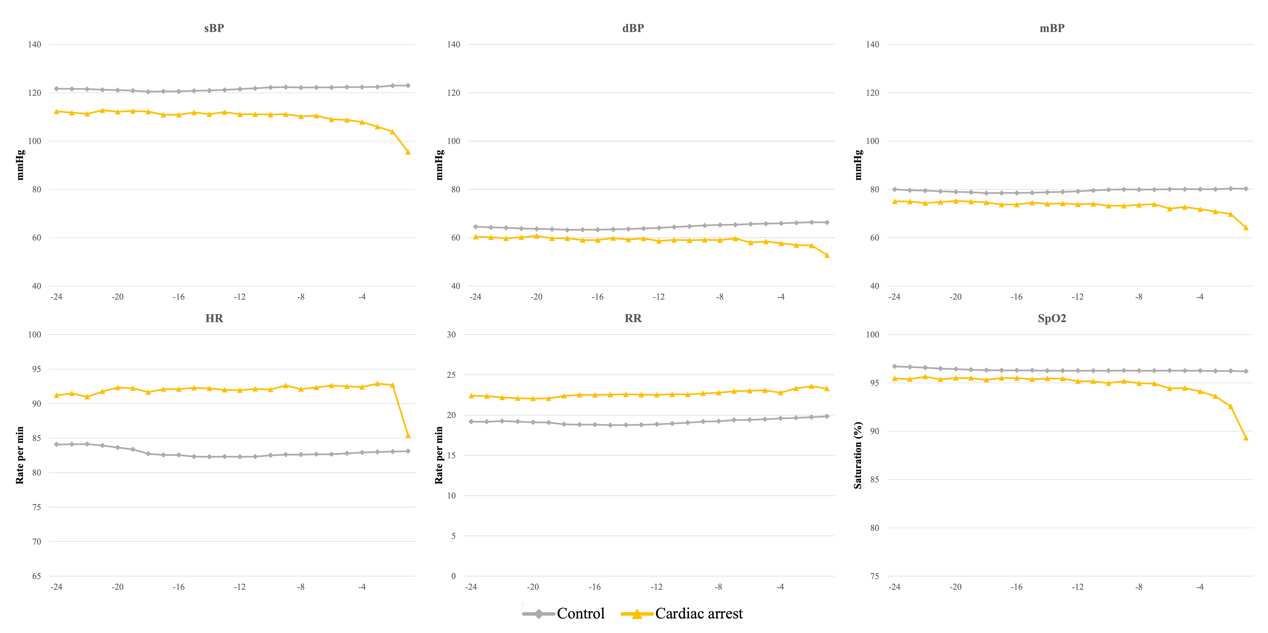


**Supplementary Figure 2B**
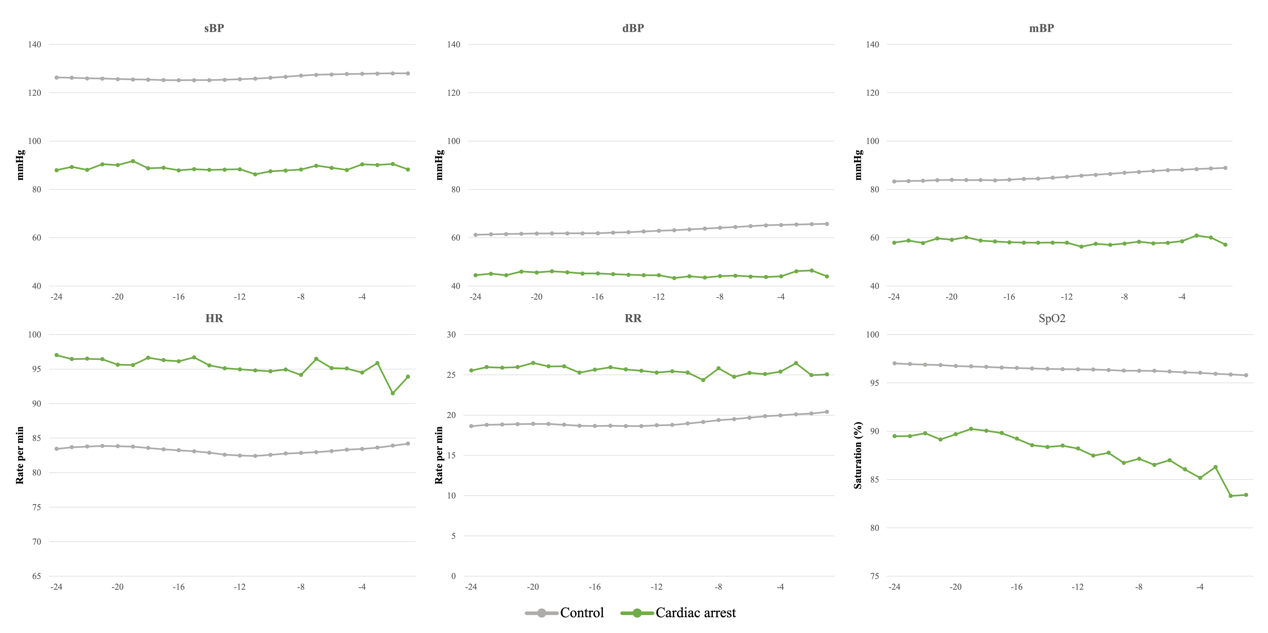


**Figure S3: Performance of the LSTM model in the MIMIC-IV database.**

AUROCs of LSTM model with vital signs as input (yellow plot), vital sign after SMOTE method as input (brown plot), and vital sign after SMOTE and 5-fold cross validation as input (orange plot) are shown. The exact values are listed in the following table. AUROC, Area of receiver operating characteristic curve; LSTM, long short-term memory; MIMIC, Multiparameter Intelligent Monitoring of Intensive Care; SMOTE, Synthetic Minority Oversampling Technique.


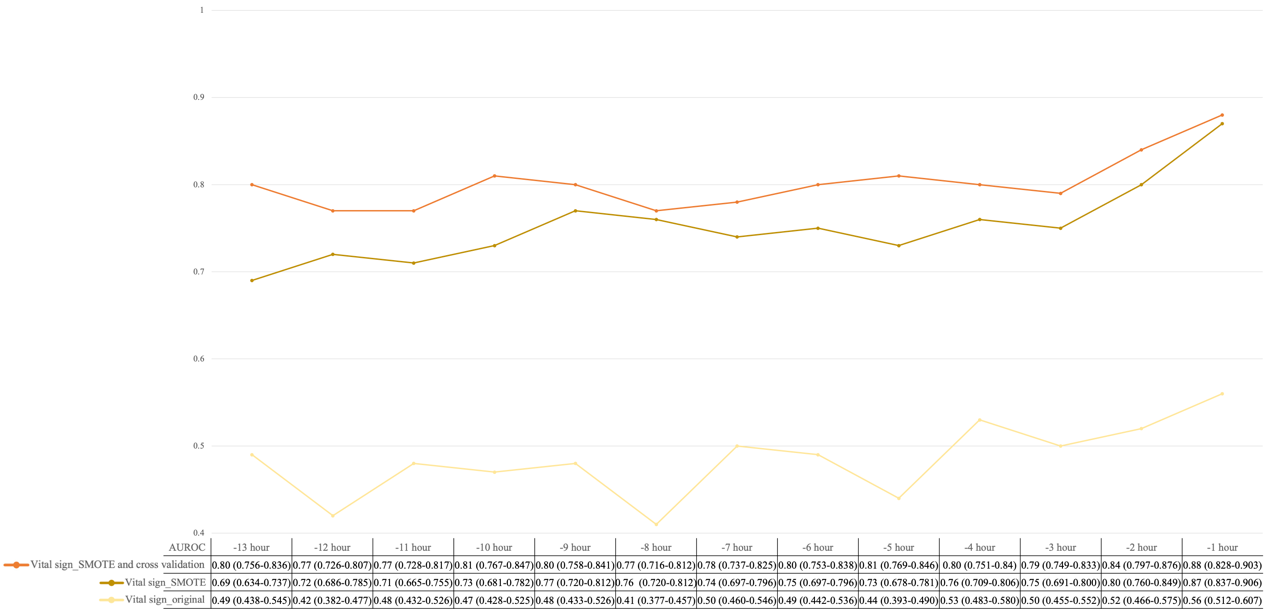


**Figure S4: Calibration of the stacked model in the MIMIC-IV cohort.**

Brier score of each specific time point by the stacked model is listed in the table above. Calibration curve for assessing the goodness of fit for the stacked model is shown below. MIMIC, Multiparameter Intelligent Monitoring of Intensive Care.


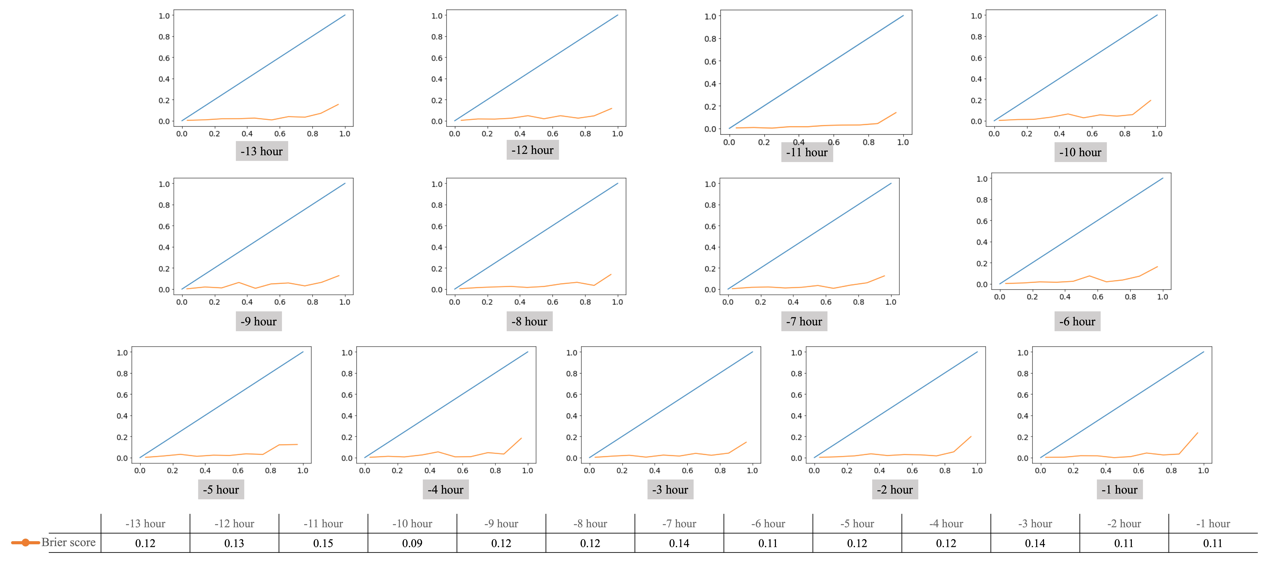


**Figure S5: Sensitivity of the stacked model with optimal and predefined cut-off in the MIMIC-IV cohort.**

Sensitivity of the stacked model using predefined 50% threshold (green plot) and optimal threshold from Youden index (red plot) are shown. The exact values are listed in the following table. MIMIC, Multiparameter Intelligent Monitoring of Intensive Care.


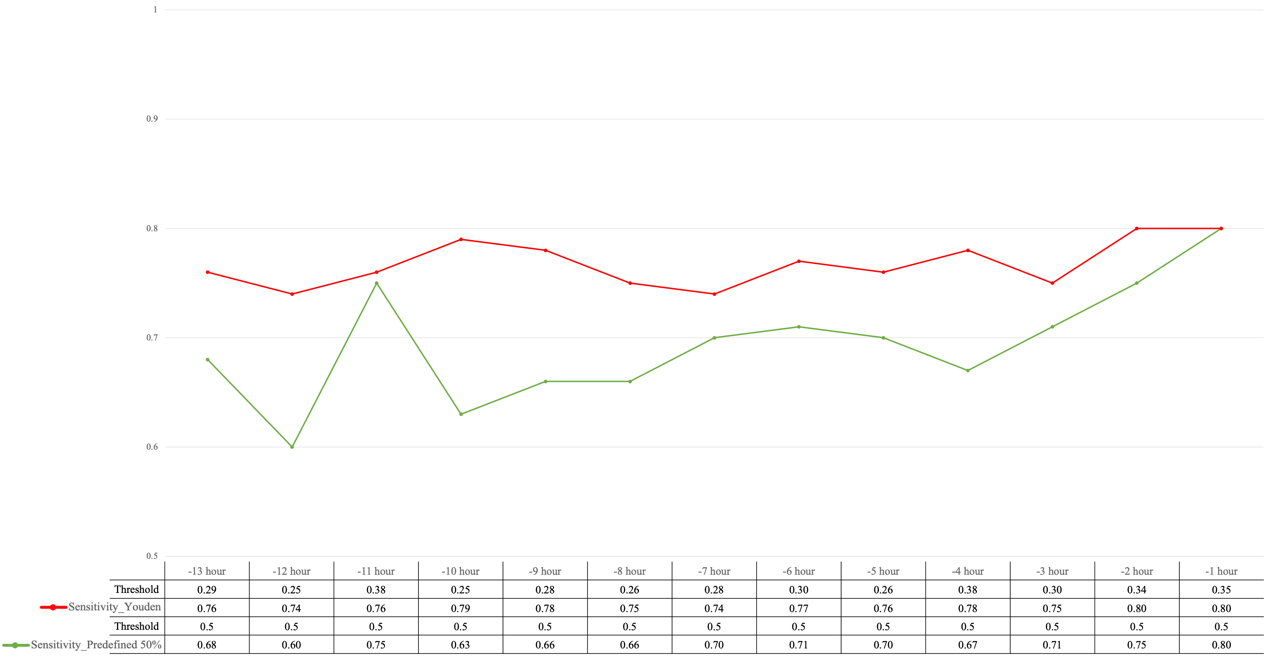


**Figure S6: SHAP value of the stacked model in the MIMIC-IV database.**

Explanation of the prediction results in each specific instance, the impact of six vital signs are displayed explicitly. Features in red raise the predicted risk higher than the average risk; features in blue keep the predicted risk lower than the average risk. (A) An example of one cardiac arrest patient in the MIMIC-IV database. (B) An example of one cardiac arrest patient in the eICU-CRD database.

**(Supplementary Figure 6A)**
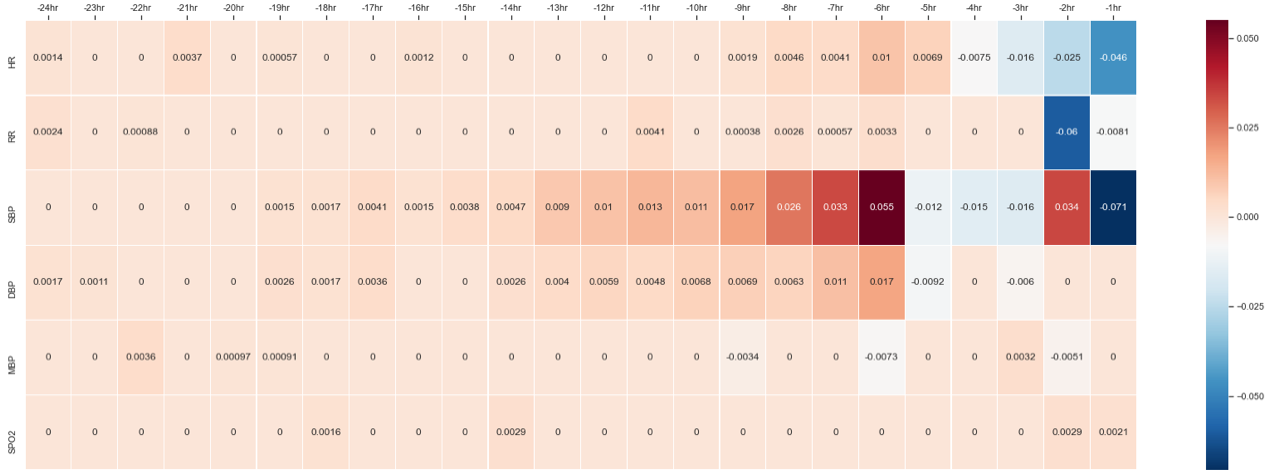


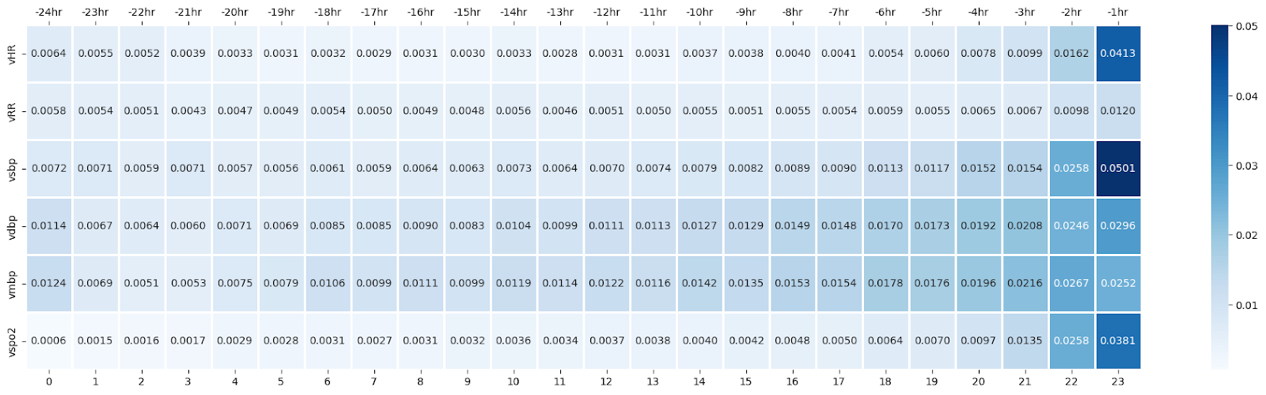


**(Supplementary Figure 6B)
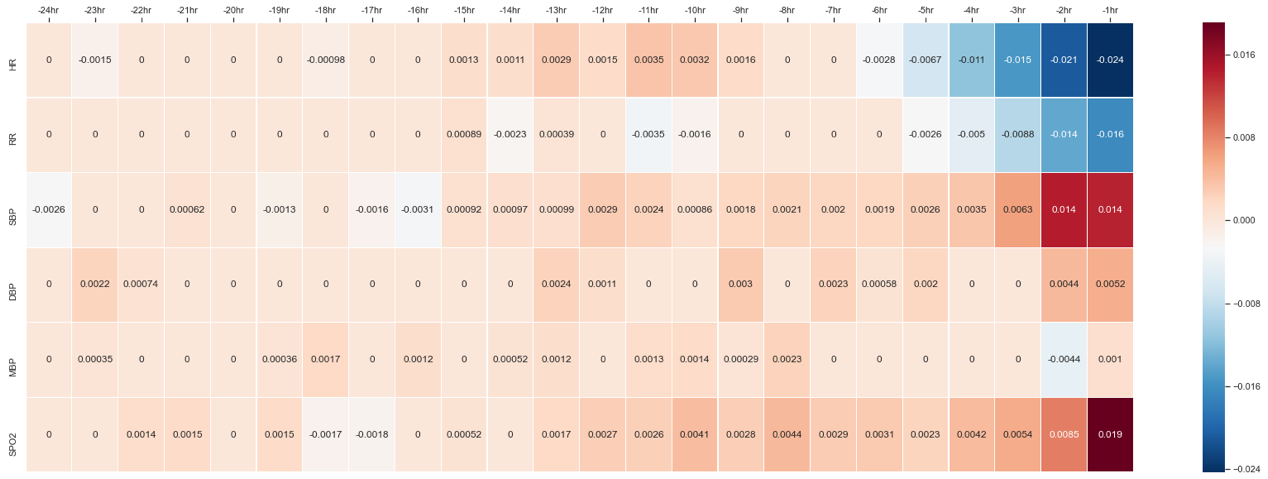
**

**
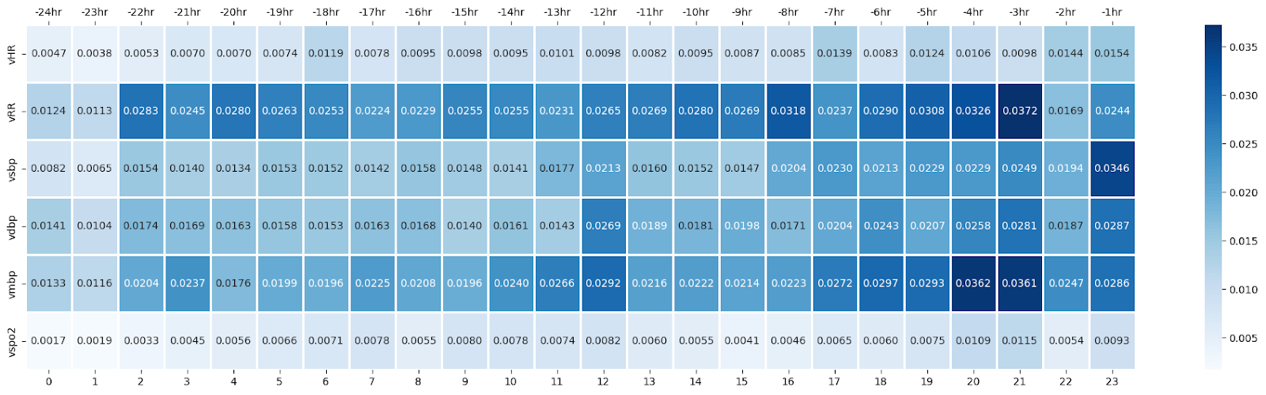
**

**Figure S7: Performance of different machine learning algorithms using the baseline features.**

AUROCs of Random Forest (purple plot), SVM (blue plot), Logistic regression (gray plot), XgBoost (green plot), and K-nearest-neighbors (red plot) using baseline features as input.

**
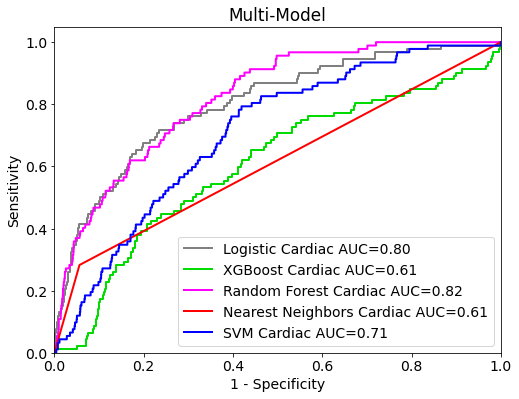
**

**Figure S8: Comparisons of the stacking results using Support Vector Machine (SVM) versus Logistic regression (LR).**

AUROC of the stacked model operated with SVM (blue plot) versus with LR (gray plot).

**
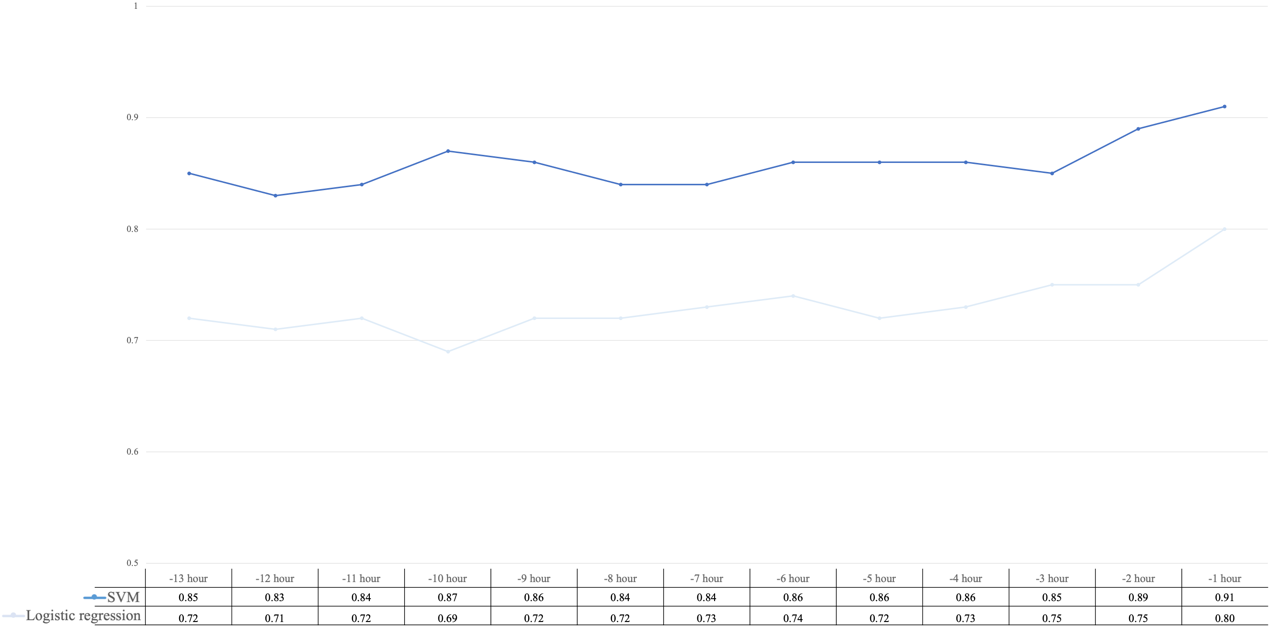
**

**Figure S9: Results of applying Neural Network in three layers of prediction.**

AUROC of applying Deep Neural Network on baseline predictions and the stacking operation (yellow plot) compared with our definite model (blue plot).


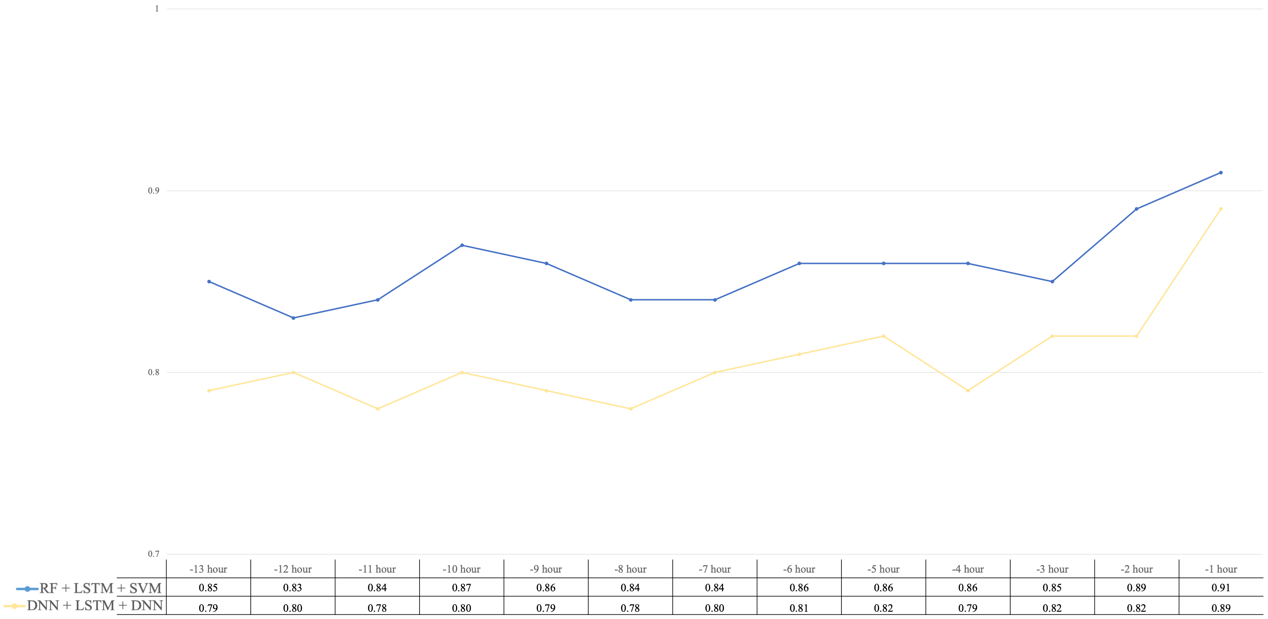


**Figure S10: Performance of the stacked model and CART model in the MIMIC-IV database.**

AUROC of the stacked model is plotted in blue; AUROC of the CART model is plotted in gray. The exact values are listed in the following table.

**
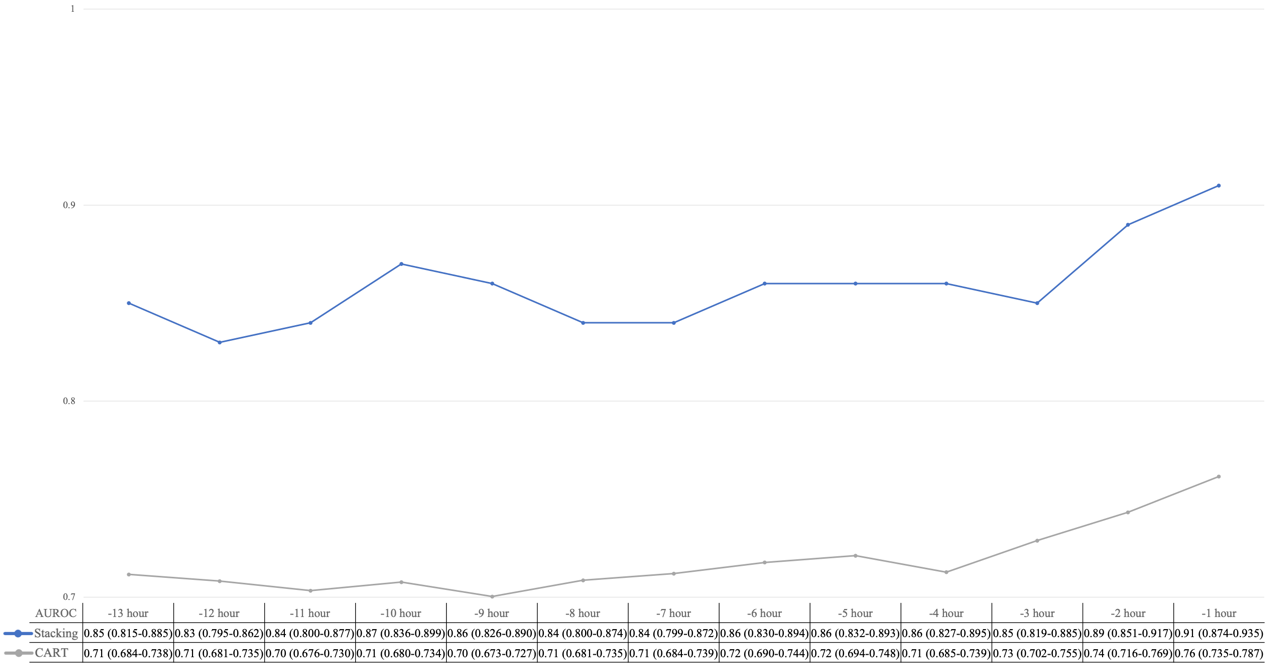
**

**Figure S11: Early warning score efficiency curve for IHCA patients in the MIMIC-IV database.**

Proportion of patients who reach a predefined threshold against the risk score sensitivity in the evaluation of IHCA. Threshold for the stacked model is a predicted probability above 50% (blue plot); threshold for the CART model is a score above 20 (gray plot). CART, Cardiac Arrest Risk Triage; IHCA, In-hospital cardiac arrest; MIMIC, Multiparameter Intelligent Monitoring of Intensive Care


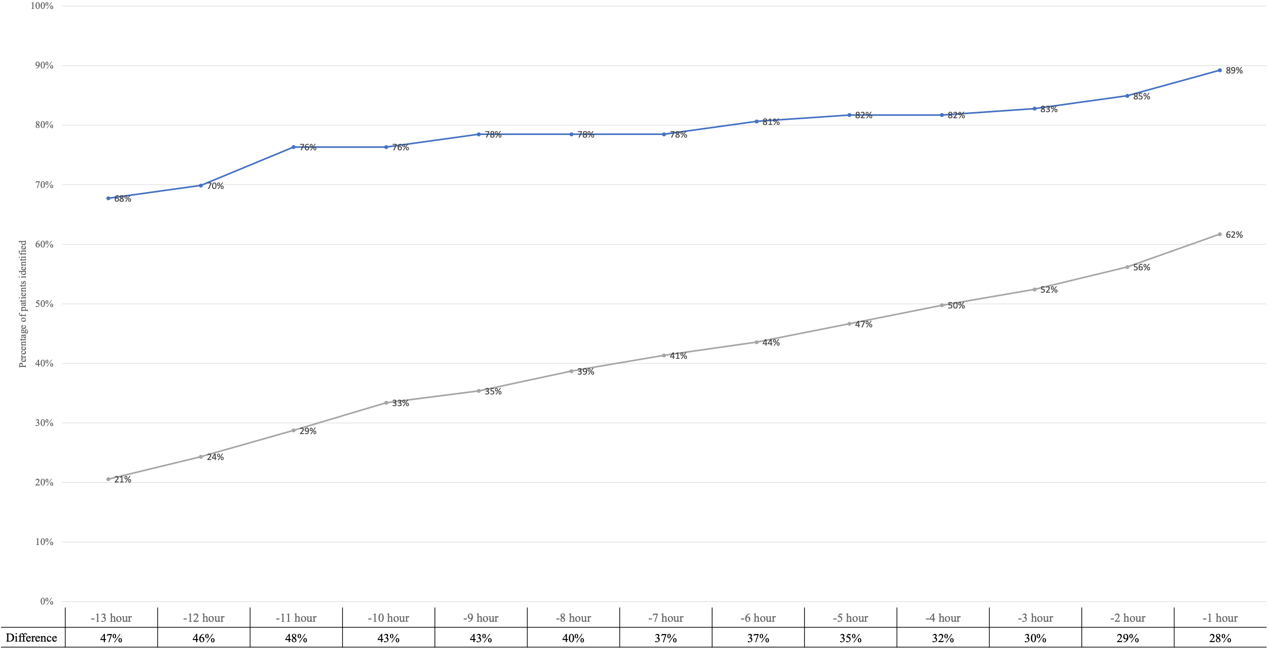


**Supplementary Material**

*Data curation and features extraction*

The following rules were used to combine multiple VS for the same hour in order to overcome the irregularity in time series. The control group had a relatively stable trend, so we calculated the mean value for all 6 VS. For the IHCA group, to maintain the deteriorating trend and exclude misleading numbers, we selected the minimum values for sBP, dBP, mBP and SpO2; maximum value if all HR data was above 80bpm and minimum value if all HR data was below 80bpm or spread across 80bpm; maximum value if all RR data was above 12/min and minimum value if all RR data was below 12/min or spread across 12/min. To achieve maximum patients in the IHCA group while maintaining the quality of data, patients were required to present with at least 1 VS measurement in the 12 hours prior to the reference time in the IHCA group, and at least 23 VS measurements in 24 hours prior to the reference time in the control group.
